# Biological activities and antioxidant potential of different biosynthesized nanoparticles of *Moringa oleifera*

**DOI:** 10.1038/s41598-022-23164-2

**Published:** 2022-11-01

**Authors:** Emad A. Shalaby, Sanaa M. M. Shanab, Walaa M. Abd El-Raheem, Eman A. Hanafy

**Affiliations:** 1 Department of Biochemistry, Faculty of Agriculture, Cairo University, Giza, 12613 Egypt; 2 Department of Botany and Microbiology, Faculty of Science, Cairo University, Giza, 12613 Egypt; 3 Department of Botany and Microbiology, Faculty of Science, Sohag University, Sohag, Egypt

**Keywords:** Biochemistry, Cancer, Chemical biology

## Abstract

The science of nanotechnology is expanding daily and has the potential to benefit people. *Moringa oleifera* is an abundant source of phenolic compounds, which are bioactive substances. It is recognised as a necessary plant because of its medicinal potential and a wide variety of health benefits. The aim of the current study is to examine the antioxidant, antibacterial, and cytotoxicity effects of five nanoparticles (La_2_O_3_, CuO, Fe_2_O_3_, Ag, and ZnO) made using bioactive chemicals in the aqueous extract of *Moringa oleifera* leaves on four human cell lines (T47D, HepG2, A549, and Wi38). The UV–visible spectroscopy analysis with a surface plasmon peak in the 300–490 nm range and the value of the zeta potential of the various biosynthesized nanoparticles ranged from + 31 to + 37 mV, indicated the repulsion between the particles and the stability of the formulation nanoparticles confirmed the formation of all nanoparticles. Additionally, the DPPH method was used to assess the antioxidant activity of five distinct metal nanoparticles. The results show that this method works in parallel and is dependent on both the concentration of NPs and the incubation time. The anticancer effect of synthesized nanoparticles against four different cell lines has been tested. The cytotoxicity assay showed a dose-dependent and time-dependent effect of nanoparticles. The obtained results conclude that acceptable potency against T47D and A549 cell lines with IC_50_ ranged from 38 to 210 μg/mL and 26 to 115 μg/mL, respectively. However, HepG2 and Wi38 cell lines showed relatively higher resistance against all tested nanoparticles when compared with Doxorubicin. Moreover, the antibacterial results revealed that silver nanoparticles exhibited the highest antibacterial activity against both *Enterococcus faecalis* and *Staphylococcus aureus*. Nanoparticles' high therapeutic activity at low concentrations opens up new avenues for the development of novel therapeutic approaches against human pathogens.

## Introduction

Because of its multiple applications as optical probes, sensors, catalysts, antibacterial agents in healing wounds, burns, and surgery applications, the synthesis of NPs has gained significant attention from the scientific community. Plants are safe natural sources that possess varied concentrations of active agents that help promoting the reduction of metallic ions and, accordingly, stabilise the nanoparticles, hence the distinctiveness of the biologically produced nanoparticles^1^.

Plant extracts are used in the green synthesis of nanoparticles (NPs). Biosynthesis of NPs is a hot research topic because it has numerous applications in various fields such as pharmaceutics, biomedicine, agriculture, and industrial fields^2,3^, which is an environment friendly approach that uses nontoxic precursors to reduce waste formation. Many plants have been used as possible precursors in the synthesis of NPs.

One of the most important medicinal plants, *Moringa oleifera Lam*. (family: Moringaceae), is largely located in the rainforest region and forest ecology but is now well-adapted to an organised cultivation system. When consumed as food, it has positive and preventive effects as well as a wide range of potent therapeutic properties, qualities with significant dietary advantages. The various plant parts of *M. oleifera*, including the leaves, flowers, fruits, seeds, and roots, are rich sources of protein, ß-carotene, and essential amino acids and minerals, as well as other phenolic compounds. Because of its extensive range of health advantages, it is regarded as a necessary plant due to its therapeutic potential. It has been discovered that the plant possesses several medicinal properties, including those that are antitumor, anti-inflammatory, antiulcer, antipyretic, antiepileptic, antispasmodic, diuretic, antihypertensive, and antidiabetic. This plant lowers cholesterol levels, strengthens cells, and has a hepatoprotective effect. Additionally, it has been employed traditionally in the regional curative system to cure cardiac issues, and the antifungal qualities are effectively used to treat a variety of illnesses^4^.

Cancer is a multi-factorial illness that stemmed from a complex fusion of environmental and hereditary determinants marked by unusual cell division. Besides, cancer is the second foremost instigator of death, which puts immense losses in the community^1^.

Metal nanoparticles (metal-NPs) have been widely studied for their antioxidant, antibacterial, anti-inflammatory, and anticancer effects. ROS are highly reactive free radicals produced during mitochondrial oxidative metabolism^5^ that contain an unpaired electron that can vitiate all macromolecules, including DNA and RNA, and cause cellular death. Plants have a sophisticated network of antioxidant metabolites and enzymes that work together to prevent oxidative damage to cellular components, protecting humans from a variety of diseases.

Metal nanoparticles are being more widely used in fields such as electronics, catalysts, medicine, and biotechnology. Because of their unique physicochemical and biological therapeutic capabilities as antibacterial and antiviral, antifungal, anti-inflammatory, and anticancer properties have been discovered. Promoting the various synthesised metal nanoparticles, silver nanoparticles have been widely used^6^.

The present work was designed to estimate the physico-chemical properties of metal NPs synthesized by *M. oleifera* aqueous extract and evaluate the biological activities of these NPs as antioxidant, antibacterial, and anticancer compared with biological standards.

## Materials and methods

### Chemicals and reagents

Pure ethanol and methanol were purchased from E. Merck Co. (Darmstadt, Germany). Sulfarhodamine, 2, 2 diphenyl-1-picrylhydrazyl (DPPH), were purchased from Sigma-Aldrich (St. Louis, MO, USA). Butylated hydroxyl toluene (BHT), Silver nitrate, zinc nitrate hexahydrate, Copper (II) nitrate trihydrate, Lanthanum Nitrate Hexahydrate, ferric chloride and Sodium hydroxide were purchased from Sigma-Aldrich (St. Louis, MO, USA). The MTT solution was purchased from BIO BASIC CANADA INC.

### Microorganisms and cancer cell lines

All microorganisms, gram positive bacteria (*Staphylococcus aureus*, *Bacillus subtilis*, and *Enterococcus faecalis*), gram negative bacteria (*Escherichia coli*, *Pseudomonas aeruginosa*, and *Salmonella typhimurium*), were obtained from the Science Faculty, Al-Azhar University, Egypt. Cell lines, normal cell lines (human lung fibroblast (WI 38)), carcinoma cell lines (human lung (A 549), breast cancer cell lines (T47D) human liver (HepG2)) were obtained from Vacsera, Giza, Egypt.

### Experimental research and field studies on plants

All Experimental research and field studies on plants, including the collection of plant material, comply with relevant institutional, national, and international guidelines and legislation.

### Collection and identification of *M. oleifera* plant

The fresh leaves of *Moringa oleifera* Lam. (Moringaceae) were collected from El-Sharqya, Egypt during June/July 2021. The plant was kindly identified and authenticated by Prof. Dr. Wafaa M. Amer, Professor of Plant Taxonomy, Faculty of Science, Cairo University, Giza, Egypt. Voucher specimens (given number Mo 1)^7^.

### Preparation of the *M. oleifera* leaves extract

Fresh, mature, healthy leaves of *M. oleifera*) were picked, repeatedly washed with tap water, double-distilled water to eliminate dust particles, and then sun dried to remove any remaining moisture. Furthermore, the dried leaves were smoothly crushed. 100 mL of double distilled water were used to rehydrate 5 g of powdered *M. oleifera* leaves. Using a stirrer-heater, the extract was heated at 60–70 °C for 60 min. or until the colour of the aqueous solution changed from watery to light yellow. The extract was filtered through Whatman filter paper (No. 1) several times. According to Rossenthaler's recommendations (1930)^8^, the extracts were then preserved for later use in sterile bottles in a refrigerator at 4 °C.

### Bio*synthesis* of silver nanoparticles (AgNPs)

Using magnetic stirring for 45 min, 90 mL of a 1 mM AgNO_3_ solution were heated at 60–70 °C. This solution received dropwise additions of 10 mL of *M. oleifera* leaf extract. The solution's colour changed from colourless to brown, confirming the reduction of Ag^+^ to AgO. Centrifugation was used to collect the synthesised AgNPs. For characterization requirements, black powder was collected, carefully assembled, and packaged^9^.

### Biosynthesis of zinc oxide nanoparticles (ZnONPs)

To synthesise ZnONPs, 1.4 g of zinc nitrate hexahydrate was dissolved in 100 mL of deionized water. 25 mL of *M. oleifera* leaf extract was dropwise added to a zinc nitrate hexahydrate (Zn (NO_3_)_2_·6H_2_O) solution (0.05 M/100 ml) heated to 60 °C with magnetic stirring. For three hours at 60 °C, the reaction mixture was continuously stirred. The dispersion's colour steadily altered over different time intervals from colourless to yellow before a white sticky precipitate was completely produced. After the reaction was finished, the mixture was centrifuged at 10,000 rpm for 10 min while being allowed to cool at 25 °C. The synthesised ZnONPs were dried for two hours at 70 °C. The paste was then collected in a ceramic crucible and dried for 4 h at 400 °C in an air-heated furnace. For physical characterization and biological applications, white powder containing ZnONPs that had completed calcination was carefully collected^9,10^.

### Green synthesis of copper oxide nanoparticles (CuONPs)

A 0.2 M aqueous solution of Copper (II) nitrate trihydrate (Cu (NO_3_)_2_·3H_2_O) was produced and kept in brown bottles. 400 ml of 0.2 M Cu (NO_3_)_2_·3H_2_O solution and 100 ml of *M. oleifera* leaf extract (1:4) were slowly combined while constantly stirring. The combination has been incubated for 24 h at room temperature. Periodically, the colour change was checked (after 30 min and 60 min). The solution was centrifuged for 15 min at 10,000 rpm when the colour of the solution changed from blue to a light brownish colour, which clearly shows the formation of CuONPs. To get rid of any contaminants, deionized water and ethanol were used to wash the resulting CuONPs. The CuONPs were then allowed to dry and grind before being used for further analysis^11^.

### Green synthesis of lanthanum oxide nanoparticles (La_2_O_3_NPs)

Plant extract was continuously stirred with 0.1 M Lanthanum Nitrate Hexahydrate for 30 min at room temperature. The lanthanum nitrate solution was stirred continuously as a 0.3 M diluted NaOH solution was added dropwise, and the mixture was then let to stand. By repeatedly washing with water and ethanol, the unreacted nitrate in the resultant precursor solution was eliminated. When the washing procedure is finished, the precursor solution's ultimate form turns from black to whitish. Filtration occurred to produce the final product^9^.

### Synthesis of Iron-oxide Nanoparticles (Fe_2_O_3_NPs)

Fe_2_O_3_NPs were created by adding 5 mL of freshly produced extract to 50 mL of ferric chloride aqueous solution (1 mM) while stirring continuously for 15 min at 50 °C to produce a brown-black solution (due to reduction). Every 30 min, 50 mL of sodium hydroxide (1 mM) was added to the mixture, and the colour of the mixture changed from brown black to black, indicating the production of colloidal Fe_2_O_3_NPs. To create solid iron-oxide pellets, the resulting colloidal solution was centrifuged at 4500 rpm for 10 min in falcon tubes after being repeatedly rinsed with distilled water using a vertex mixer. The water supernatant was then decanted out of the pellets. After washing, the Fe_2_O_3_NPs was dried in an oven for two hours at 80 °C^9^.

### Characterization of nanoparticles (NPs-Me)

#### UV–vis spectrophotometric analysis

Initially, periodic reaction solution sampling has been used to examine the reaction medium's colour change, and the UV–VIS absorption of the solution was used to confirm NPs production. According to Khattak et al*.*^9^ the reaction mixture aliquots were examined using a UV–visible spectrophotometer at 200 and 800 nm.

#### Fourier Transform Infrared (FTIR) spectroscopy

FTIR analysis was done for different biosynthesized NPs with a Shimadzu FTIR spectrometer at room temperature over the range of 400−4000 cm^−1^ at a resolution of 3 cm^−1^ in KBr pellets.

#### X-ray diffraction (XRD)

XRD data of the *M. oleifera* and NPs were collected with a D8 Advance with DAVINCI design (Bruker, Germany), using X-ray source the CuKα radiation (wavelength λ = 1.5418 Å), at 40 kV and 40 mA, a 2θ range of 20–80°, a step size of 0.02°, and a time/step of 0.6 s. A Si zero-background sample holder was used, operated by DIFFRAC. Measurement Centre Version V7.3.0 (32Bit) software, while the assignment of peaks was based on the Powder Diffraction Files (PDF) of the COD database (Crystallography Open Database).

#### Zeta potential

Zeta-potential (by Zeta Compact, CAD) was used for the assessment of surface charges of different formed nanoparticles.

#### Transmission electron microscopy (TEM)

The shape, size, and microstructures of the NPs obtained were analysed by transmission electron microscopy (Model JEM 3100 LV, JOEL, USA).

### Biological activities

#### DPPH radical scavenging activity

The method of Yen and Chen^12^ was used to measure the scavenging effects of various metal and metal-NPs biosynthesized by *M. oleifera* leaf extracts. A 2.0 mL aliquot of sample at 100 and 200 μg mL^−1^ was added to a test tube containing a 0.16 mM DPPH solution (in methanol). The mixture was vortexed for one minute before being left at room temperature in the dark for 30 min. At 517 nm, the absorbance of each sample solution and BHT, a synthetic standard, was measured. The following formula was used to determine the scavenging activity percentage:

$$\begin{gathered} {\text{Inhibition(\% ) = [(A}}\;{\text{control} - {A}}\;{\text{sample)/A}}\;{\text{control]}} \times {100 } \hfill \\ \hfill \\ \end{gathered}$$

where A_control_ was the absorbance of DPPH. A_sample_ was the absorbance of the sample.

### Cytotoxic activity

Cytotoxic activity of all biosynthesized NPs by *M. oleifera* leaves extracts were determined by the MTT protocol according to Slater et al*.*^13^.

### a-Cell culture

T47D, HepG2, A549, and Wi38 were obtained from the Vacsera (Giza, Egypt). Cells were maintained in RPMI-1640 supplemented with 100 µg/mL streptomycin, 100 units/mL penicillin and 10% heat-inactivated fetal bovine serum in a humidified 5% (v/v) CO_2_ atmosphere at 37 °C.

### b-Cytotoxicity assay

To create a full monolayer sheet, 1 × 10^5^ cells/ml (100 μl/well) of cells were added to the 96-well tissue culture plate. The plate was then incubated at 37 °C for 24 h. Following the formation of a confluent sheet of cells, growth media was decanted from 96 well micro titre plates. With wash media, the cell monolayer was washed twice.

The tested material was diluted three times in RPMI medium with 2% serum (maintenance medium), and then the wells were examined with 0.1 ml of each dilution, leaving three as controls and receiving only maintenance media.

The plates were examined after 37 °C incubation. Each well received a 20 μl MTT solution (5 mg/ml in PBS), which was thoroughly mixed with the media by shaking at 150 rpm for 5 min, and the MTT metabolite was incubated at 37 °C with 5% CO_2_ for 4 h.

The media was discarded. (If required, dry the plate on paper towels to remove residue). In DMSO, 200 μl of formazan (a MTT metabolic product) were dispersed. The solvent and formazan were shaken together vigorously for five minutes at 150 rpm. At 620 nm, the background was removed and the optical density was read. Optical density and cell quantity should be directly correlated.

### Antibacterial activity (Sensitivity tests)

A modified Kirby-Bauer disc diffusion method was used to evaluate the antibacterial activity of the samples that were tested^14^. In a nutshell, 10 ml of freshly prepared media were used to cultivate 100 μl of the test bacteria until they achieved a count of 10^8^ cfu/ml^15^. On to agar plates corresponding to the broth in which the bacteria were kept, 100 μl of the bacterial suspension was applied. Each organism's isolated colonies should be chosen from primary agar plates and examined for susceptibility using the disc diffusion method^16,17^.

Gram positive and negative bacteria were cultured for 24–48 h at 35–37 °C. Gram positive bacteria included *Staphylococcus aureus*, *Bacillus subtilis*, and *Enterococcus faecalis*. Gram negative bacteria included *Escherichia coli*, *Pseudomonas aeuroginosa*, and *Salmonella typhimurium*. The inhibitory zones' sizes were measured in millimetres^14^. Filter discs impregnated with 10 μl of solvent (DMSO) were employed as a negative control, whereas standard discs containing the antibacterial agent’s ampicillin and kanamycin were utilised as positive controls for antibacterial activity. Blank paper discs (Schleicher & Schuell, Spain) with an 8.0 mm diameter were impregnated with 10 μl of stock solutions.

### Statistical analysis

Values are analysed as mean SE or SD. Statistical analysis was done utilising the "costat" statistic computer program. Statistical analysis was established on one-way analysis of variance (ANOVA), followed by the student-Newman Keuls test, and least significant difference (LSD) at *P* < 0.05.

## Results and discussion

### Synthesis and characterization

When the aqueous extract of *Moringa oleifera* was added to each of the metal solutions (La_2_O_3_, CuO, Fe, Ag, and ZnO), pH was adjusted, and the solution was heated. The colour of the reaction was formed immediately and started to be converted gradually from colourless to brown. Within minutes, the intensity of the brown colour increased rapidly with time and remained stable within one hour. It is well known that metalNPs have a brown colour due to their characteristic excitation of surface plasmons in the range of 300–490 nm^18^. Therefore, a transition of the solution from colourless to brown indicates the synthesis of Ag-NPs^19^.

**Figure 1:**
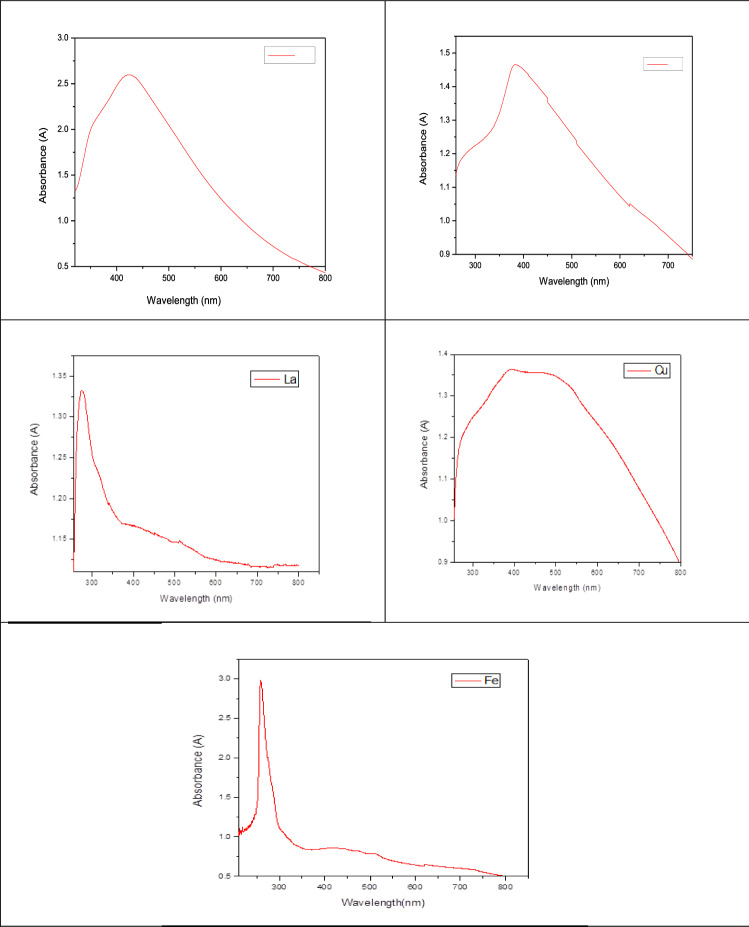
UV–VIS spectra of biosynthesized nanoparticles from *M. oleifera.*

This result means that the aqueous extract of *M. oleifera* has a high reduction potential for reduced metal ions and the formation of metal-NPs. The UV–VIS spectra of synthesised metalNPs demonstrated the maximum peak at 300, 370, 290, 420, and 390 nm for La_2_O_3_, CuO, Fe_2_O_3_, Ag, and ZnO, respectively, as shown in Fig. 1. Similar surface plasmon resonance (SPR) peaks were observed in many studies of green synthesis for silver NPs , as reported by several studies:^20–22^ Also, according to the earlier study by Alsammarraie et al*.* (2018)^2^, NPs explicated well-known peaks approximately at 350–450 nm. The accomplished outcomes are in concurrence with the results reported elsewhere.

FTIR measurements were carried out to identify the promising biomolecules in the *M. oleifera* aqueous extract accountable for the La_2_O_3_, CuO, Fe, Ag, and ZnO ion reduction and also the capping agent liable for the reduced metal-NPs stability.

**Figure 2:**
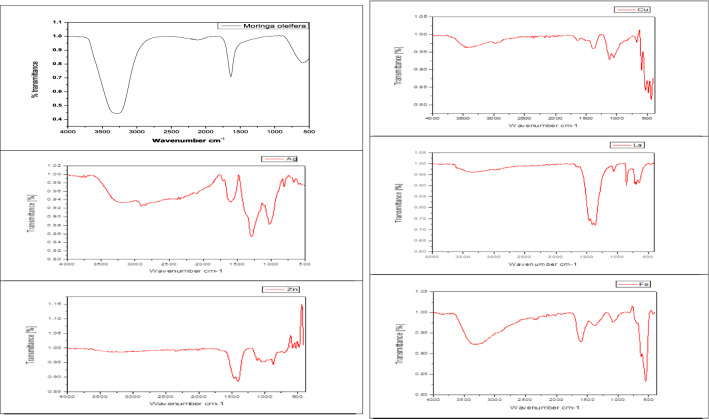
FTIR spectrum of biosynthesized nanoparticles from *M. oleifera.*

As shown in Fig. 2, the FTIR spectra of aqueous extract and different metal-NPs were recorded in the frequency range between 4400 and 350 cm^−1^ in the mode of % transmittance (%T). It was shown that there were slight shifts in the FTIR peaks of *M. oleifera* extract (3330 and 1652 cm^−1^) and the synthesised La_2_O_3_ (1550 and 1440 cm^−1^) but in case CuO (1450 and 1265 cm^−1^) and the synthesised Fe (3330 and 1652 cm^−1^). However, 1633, 1351, and 1001 cm^−1^ peak was recorded in the case of nano silver. The absence of some peaks, particularly 3330 and 1652 cm^−1^, in the synthesised metal NPs compared to the aqueous extract, as well as the slight shifts in the peaks, suggest that some functional groups are involved in the bioreduction steps or processes. The bands from 3455 up to 3383 cm^−1^ in the FTIR spectra correspond to O–H stretching vibrations, which indicates the presence of alcohol and phenol. It was reported that hydroxyl groups (O–H) have stronger binding abilities with metal ions. This suggests the presence of various functional groups responsible for the reduction of various metal ions to the NPS form. The FT-IR analysis suggested that the reasonable mechanism of metal-NPs formation may be due to the reduction of metal^+^ ions that takes place together with oxidation of different bioactive compounds such as phenolic components of polyols or other reducing components in plant extract^1,23^. Also, Jacob et al*.*^24^) found that the general observation advocates the intentness of antioxidants such as flavonoids and phenolic compounds as reducing agents, and proteins may act as coating or stabilizing agents.

**Figure 3:**
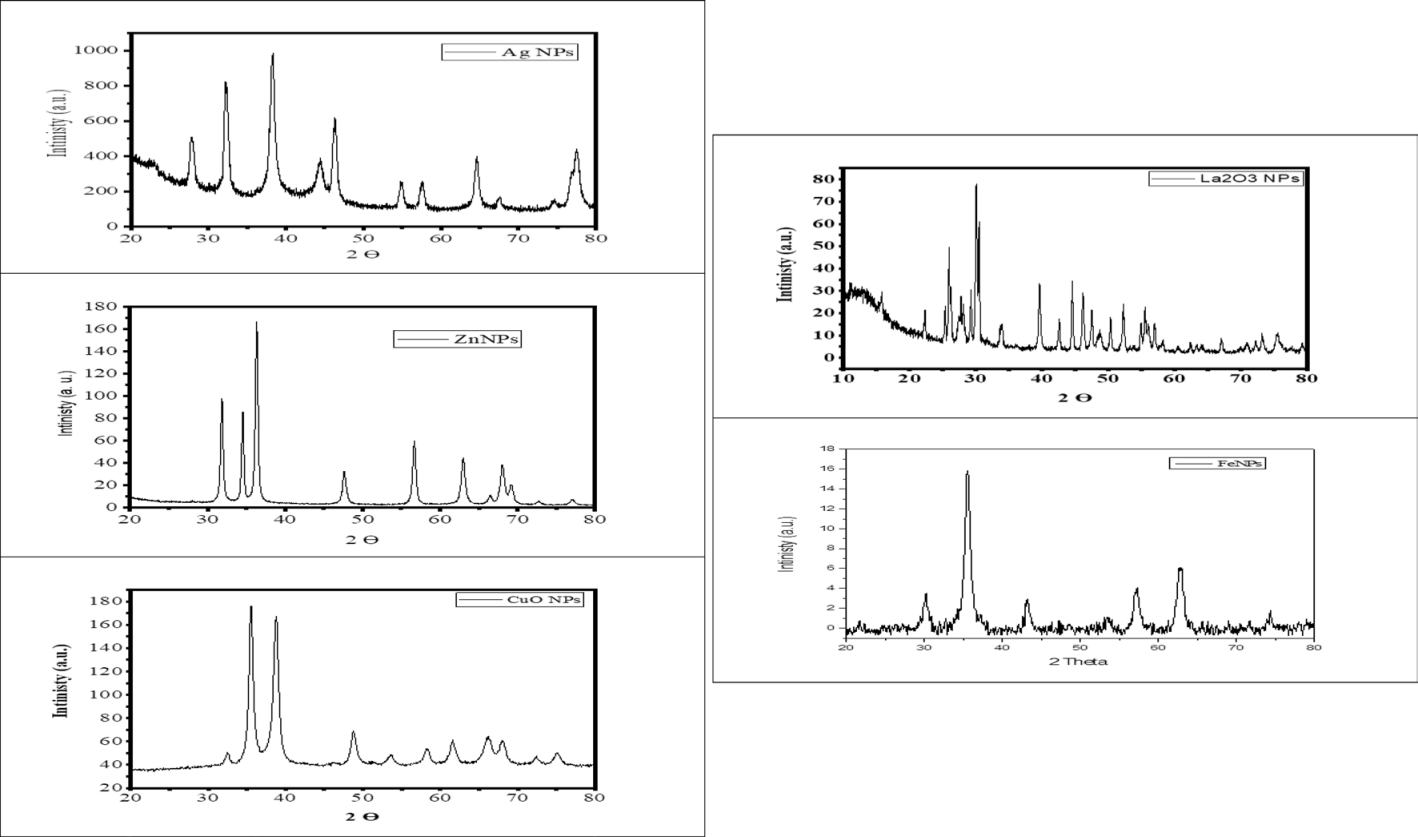
X-ray diffraction pattern of various metal nanoparticles from aqueous extract of *M. oleifera.*

Figure 3 shows the XRD pattern of the prepared NPs. In the XRD pattern of silver, ten diffraction peaks were observed at 2θ = 27.85°, 32.0°, 38.2°, 44.5°, 47.3°, 55.0°, 57.8°, 66.1° and 77.2°, and seven diffraction peaks were observed with zinc NPs as 2θ = 33.2°, 35.0°, 37.6°, 48.4°, 57.0°, 63.9°, 68.5°. Cupper NPs gave 6 diffraction peaks as 35.1°, 38.4°, 49.1°, 63.2°. However, 15 different diffraction peaks were recorded with La NP as shown in Fig. 3.

All diffraction peaks are in good agreement with the standard value (JCPDS card No. 04-0783). The obtained results of the zeta potential of the various biosynthesized NPs (La_2_O_3_, CuO, Fe, Ag, and ZnO) using *M. oleifera* aqueous extract revealed that the zeta ranged from + 31 to + 37 mV. It is well known that the value of ζ-potential gives us predictive information on the stability of the formed NPs. The little thing about the zeta value is that it is of high stability because it is greater than the positive 30 mV or less than the negative 30 mV. It is noted that the obtained values exhibit positive values due to the positive surface charge of aqueous extract owing to the presence of –NH_2_ groups along the backbone of the biomolecular structure, which causes a larger repulsive force. Consequently, enhancement of the stability against agglomeration can be predictable^25^.

**Figure 4:**
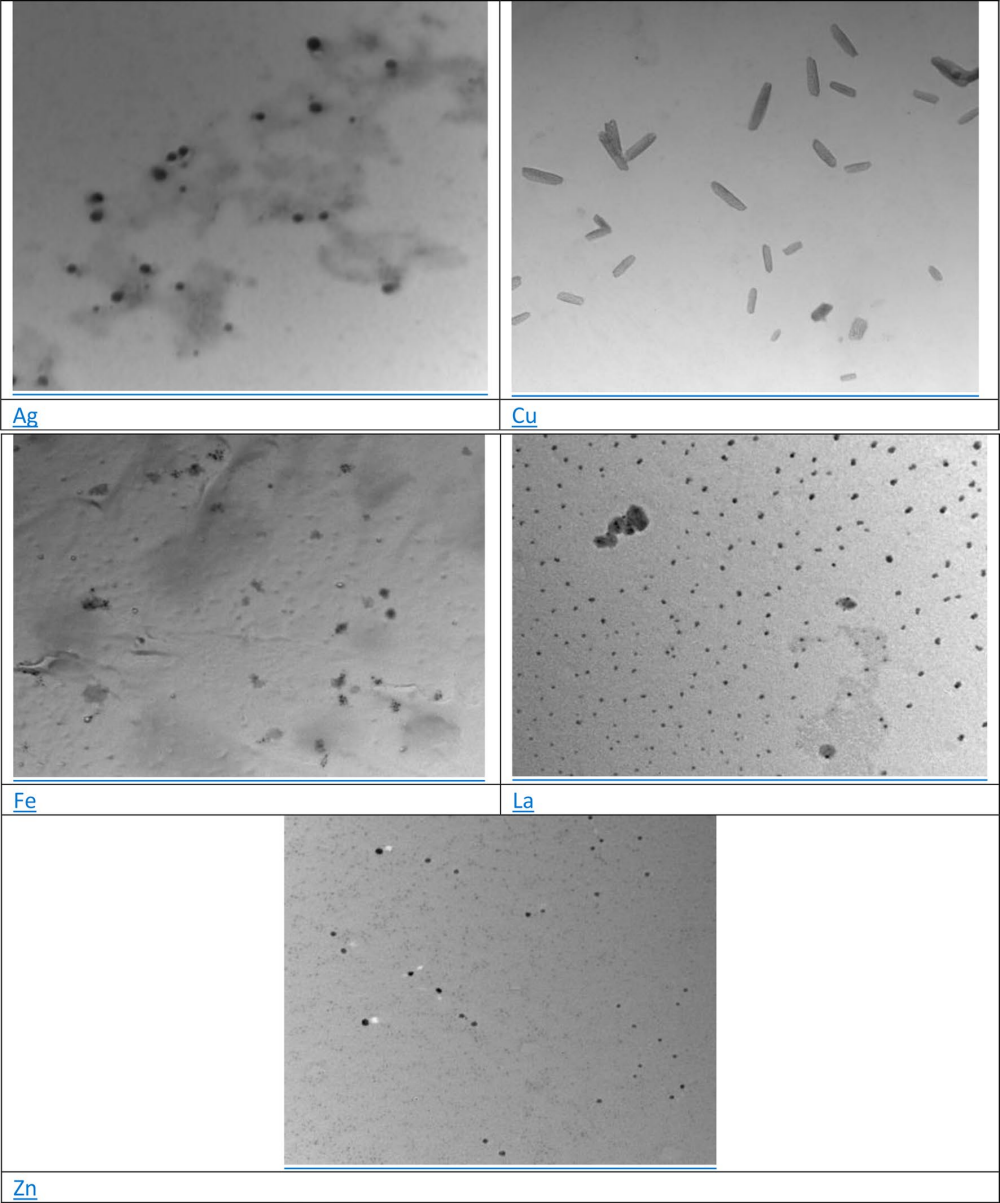
TEM images of the synthesized metal nanoparticles (La_2_O_3_, CuO, Fe_2_O_3_, Ag, and ZnO).

Transmission electron microscopy images of ZnO NPs of two shapes are spherical and hexagonal in size range of 57 nm, However, Circular nanoparticles with a size ranged 35–40 nm were determined with La-NPs. Moreover, produced Cu-NPs are hemispherical in shape with different diameters in the range 11–21 nm. the biosynthesised Fe NP have irregular spherical and porous morphology with 66 nm, but the silver nanoparticles were in spherical shape with varying sizes ranging from 50 to 70 nm (Fig. 4). These results agree with previous studies by Tippayawat et al*.*^26^ who reported spherical Ag-NPs synthesized using Aloe vera extract between 70 and 190 nm in size. Also, the same results with copper oxide were observed by Bagherzadeh^3^.

### Biological activities

#### Antioxidant activity

There are different methods and assays used for determining the antioxidant activity of metal nanoparticles. The most common method used is the DPPH assay due to its wide application to determine the free radical scavenging effect of different antioxidant agents. The DPPH possesses scavenging abilities due to the presence of the hydrogen or electron-donating activities of antioxidant agents. When DPPH results were investigated, it was observed that antioxidant activity had increased in a dose-dependent manner^27^.

The antioxidant activity of five different metal NPs was evaluated using the DPPH radical scavenging method. The results, which are shown in Table 1, show that this method works in parallel and is dependent on both the concentration of NPs and the incubation time.

**Table 1:** Antioxidant activity (%) of different metal nanoparticles against DPPH at 100 and 200 μg mL^−1^.

M-NPs	Concentration (μg mL^−1^)	
	100	200
La_2_O_3_	61.0 ± 3.5	78.5 ± 3.9
CuO	72.5 ± 4.1	82.0 ± 1.5
Fe_2_O_3_	65.8 ± 1.4	69.09 ± 2.6
Ag	78.37 ± 2.4	88.6 ± 3.7
ZnO	59.3 ± 1.4	67.8 ± 2.8
BHT	89.4 ± 1.4	92.8 ± 3.2

Data are given as mean ± SE (n = 3). M-NPs: Metal nanoparticles.

The obtained results showed that silver NPs recorded the significantly highest antioxidant activity against DPPH radical assay by 78.37 ± 2.4 and 88.6 ± 3.7% at 100 and 200 µg/ml respectively, during 30 min of incubation as shown in Table 1, followed in descending order by copper oxide NPs by 72.5 ± 4.1 and 82.0 ± 1.5% at 100 and 200 µgml^−1^ respectively, and by iron NPs and compared with BHT as a synthetic standard, which recorded the highest percentage of antioxidant against DPPH radicals by 89.4 ± 1.4 and 92.8 ± 3.2% at 100 and 200 µg/mL, respectively. These results agreed with the results obtained by Sahyon and Al-Harbi^25^, who mentioned that the green synthesised NPs showed high antioxidant activity compared with standard ascorbic acid. The obtained results agree with the hypothesis that the loading of green extract on the NPs will increase their antioxidant capacity and, hence, may inhibit the occurrence of lipid peroxidation inside the cell.

Furthermore, Kokila et al*.*^1^ that the observed antioxidant activity of AgNPs might be due to the existence of an assortment of phytochemicals like phenolics, flavonoids, and other active ingredients on the surface as capping agents on the NPs. AgNPs exhibited lower IC_50_ values in the DPPH radical assay and better antioxidant activity than plant extracts due to their smaller size and stability.

#### Cytotoxic effects

**Figure 5:**
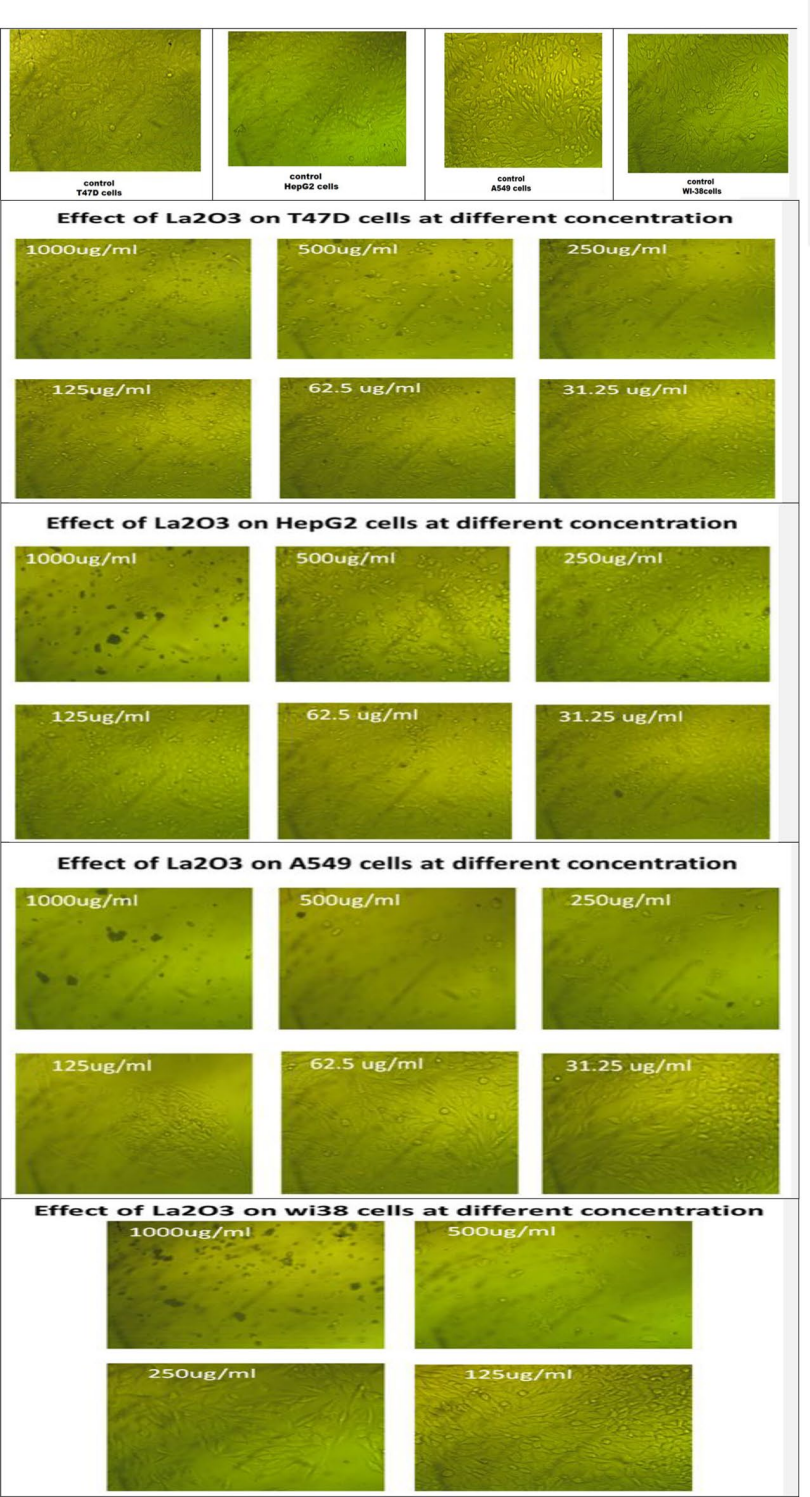
Images of treated cell lines with different biosynthesized nanoparticles compared with untreated cell lines (control).

**Figure 6:**
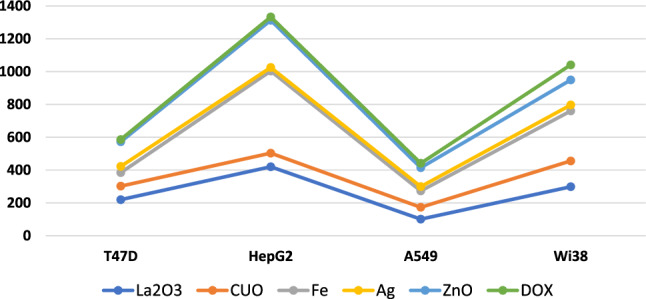
IC_50_ of different biosynthesized nanoparticles against various cancer cell lines.

The MTT assay was used to assess the cytotoxic properties of biosynthesized NPs (La_2_O_3_, CuO, Fe_2_O_3_, Ag, and ZnO) from *Moringa oleifera* against four different tumour cell lines. Different cell lines were used according to their origin and morphology, as well as sensitivity and receptor site behaviour. The cytotoxicity was calculated as a percentage, IC_50_, and a selective index (SI). The obtained results of the NPs showed acceptable potency against T47D and A549 cell lines with an IC_50_ range of 38 to 210 μg/mL and 26 to 115 μg/mL, respectively. However, HepG2 and Wi38 cell lines showed relatively higher resistance against all tested NPs with an IC_50_ of 21 to 419 μg/mL and 36 to 304 μg/mL respectively, when compared with Doxorubicin (DOX) as a standard anticancer drug (with an IC_50_ = 13.73, 20.09, 26.86, and 92.05 µg/ml against T47D, HepG2, A549, and Wi38, respectively) as shown in Tables 2, 3 and Figs. 5, 6. This means that the cytotoxicity pattern of the tested NPs on both T47D and A549 cell lines is similar, while it differs on HepG2 and Wi38. These results indicate that the effect of different NPs on all tested cell lines (four cell lines) is concentration dependant through the concentrations tested (31.25–1000 μg/mL). However, high cytotoxic activity was noticed with AgNPs at low concentrations (from 62.5 μg/mL) after which the effect was very strong, hence most of the cells died at about 250 μg/mL. This effect can be explained as receptor independent for these types of cells^28^. These results agreed with the results obtained by Almessiere et al*.*^29^, who mentioned that NPs have broad-spectrum anti-cancer properties. The activities of NPs are determined by (i) size and surface area, (ii) morphology, (iii) concentration/dose, (iv) exposure time, and (v) surface charge dispersion. Furthermore, Almessiere et al*.*^30^ found that the cytotoxic effects of NPs also govern tumour development. The release of Ag^+^ ions by silver NPs in tumour cells causes cytotoxicity. Following oxygen reduction by an electron from the electron transport chain, NPs facilitate the generation of ROS (Reactive Oxygen Species) and superoxide in mitochondria. Excessive ROS causes oxidative damage to cell components such as DNA, proteins, and lipids, ultimately leading to cell death. When cancer cells are treated with NPs, the nucleus may disintegrate and fragment, resulting in cancer cell death.

**Table 2:** Anticancer activity of different biosynthesized nanoparticles from *M. oleifera*.

Treatments	Conc. (μg/ml)	Toxicity		Percentage	
		T47D	HepG2	A549	Wi38
La_2_O_3_	31.25	2.00 ± 1.84	1.63 ± 2.70	1.02 ± 1.68	0.67 ± 2.90
	62.5	1.68 ± 1.94	2.97 ± 2.30	14.46 ± 1.39	2.46 ± 3.77
	125	4.44 ± 1.94	0.48 ± 2.27	70.63 ± 1.35	1.59 ± 1.55
	250	64.88 ± 3.39	20.56 ± 1.96	87.02 ± 1.12	57.09 ± 2.29
	500	83.17 ± 0.72	63.80 ± 2.29	97.00 ± 0.08	84.95 ± 1.22
	1000	93.45 ± 0.70	93.78 ± 1.08	97.22 ± 0.17	97.34 ± 0.10
CuO	31.25	2.65 ± 1.83	1.77 ± 2.65	4.71 ± 1.88	0 ± 3.46
	62.5	50.49 ± 1.31	48.11 ± 0.55	57.37 ± 1.00	0.31 ± 2.58
	125	76.19 ± 1.95	77.04 ± 4.41	90.02 ± 0.61	48.13 ± 1.79
	250	95.83 ± 0.52	94.69 ± 1.57	97.17 ± 0.08	85.25 ± 3.03
	500	96.81 ± 0.26	97.37 ± 0.02	97.45 ± 0.08	94.67 ± 0.77
	1000	96.16 ± 0.76	97.61 ± 0.05	97.11 ± 0.17	97.13 ± 0.11
Fe_2_O_3_	31.25	0.70 ± 1.18	0.91 ± 2.42	0.23 ± 1.49	0.26 ± 2.24
	62.5	8.50 ± 1.46	1.00 ± 2.80	16.84 ± 1.38	0.51 ± 4.06
	125	47.13 ± 1.53	3.30 ± 3.32	71.03 ± 1.75	6.91 ± 1.38
	250	77.33 ± 2.73	4 50 ± 1 49	94.10 ± 0.78	48 44 ± 2 20
	500	94.75 ± 0.57	53.28 ± 0.85	97.00 ± 0.18	85.66 ± 0.54
	1000	95.29 ± 0.67	88.95 ± 0.80	97.22 ± 0.10	97.49 ± 0.23
Ag	31.25	51.35 ± 1.00	48.97 ± 1.99	64.80 ± 2.21	47.26 ± 0.91
	62.5	89.50 ± 0.93	78.38 ± 0.78	93.76 ± 0.56	82.28 ± 1.04
	125	96.70 ± 0.25	97.27 ± 0.27	96.77 ± 0.03	96.31 ± 0.69
	250	96.75 ± 0.15	97.37 ± 0.16	96.83 ± 0.18	97.44 ± 0.18
	500	97.08 ± 0.06	97.32 ± 0.10	97.17 ± 0.05	97.44 ± 0.14
	1000	97.02 ± 0.16	97.37 ± 0.10	97.39 ± 0.13	97.29 ± 0.06
ZnO	31.25	0.54 ± 3.19	1.43 ± 1.71	4.82 ± 1.11	0.20 ± 2.68
	62.5	2.65 ± 2.01	1.43 ± 2.51	29.93 ± 3.69	1.08 ± 2.77
	125	40.37 ± 0.91	4.83 ± 2.41	52.95 ± 0.88	52.94 ± 1.97
	250	96.05 ± 0.33	48.78 ± 0.94	70.12 ± 0.81	86.43 ± 1.86
	500	96.37 ± 0.40	96.17 ± 0.28	87.30 ± 1.37	97.03 ± 0.31
	1000	97.08 ± 0.12	97.18 ± 0.18	97.00 ± 0.18	97.39 ± 0.21
Drug (Doxorubicin)	31.25	51.41 ± 1.72	50.45 ± 1.18	46.03 ± 2.36	4.66 ± 2.35
	62.5	73.32 ± 1.53	69.01 ± 2.14	73.02 ± 2.49	26.32 ± 3.49
	125	85.44 ± 0.27	83.31 ± 1.72	86.79 ± 1.83	75.42 ± 1.67
	250	94.37 ± 0.72	90.63 ± 2.46	96.94 ± 0.12	88.43 ± 0.86
	500	97.13 ± 0.11	97.37 ± 0.14	97.17 ± 0.17	96.72 ± 0.69
	1000	97.19 ± 0.19	97.61 ± 0.17	97.28 ± 0.12	97.39 ± 0.06

**Table 3:** Selectivity index (SI) of different biosynthesized nanoparticles from *M. oleifera*.

Treatments	Cell lines		
	T47D	HepG2	A549
Drug (Doxorubicin)	6.70	4.58	3.43
La_2_O_3_	1.36	0.71	2.96
CuO	1.91	1.89	2.18
Fe_2_O_3_	1.87	0.61	3.06
Ag	0.96	1.69	1.38
ZnO	1.02	0.53	1.32

SI = IC_50_ no cancer cell/IC_50_ cancer cell.

Also, the cytotoxicity increases with green synthesised NPs concentration, suggesting their use as an alternative therapeutic agent. Moreover, NPs are also known to boost nucleic acid (DNA) repair in cells, thus blocking the growth of tumour cells. Moreover, the active ingredients and antioxidants of plant extracts on the surface of NPs also play a protective role against oxidative stress-related diseases such as tumours and inflammation^31^. Other study by Ávalos et al*.*^32^ and Taghavizadeh Yazdi et al*.*^33^ revealed that the cytotoxic effects of silver nanoparticles are related to interactions with cellular functional proteins that eventually lead to cellular changes. The shape, size and surface charge of metal nanoparticles play a vital role in this action. Also, Farhangi et al*.*^34^ concluded that the synthesized CuO/CeO_2_ NC showed cell toxicity properties towards breast cancerous cell lines (MCF-7) in a dose and time-dependence manner, while the toxicity of CuO/CeO_2_ NC was significantly lower on normal fibroblastic cells.

#### Antibacterial activity

When a filter paper disc impregnated with a tested chemical is placed on agar, the chemical will diffuse from the disc into the agar. This diffusion will place the chemical in the agar only around the disc. The solubility of the chemical and its molecular size will determine the size of the area of chemical infiltration around the disc. If an organism is placed on the agar, it will not grow in the area around the disc if it is susceptible to the chemical. This area of no growth around the disc is known as a "zone of inhibition" or "clear zone". For the disc diffusion, the zone diameters were measured with slipping callipers by the National Committee for Clinical Laboratory Standards^16^.

Agar-based methods such as Etest and disc diffusion can be good alternatives because they are simpler and faster than broth-based^35,36^.

**Figure 7:**
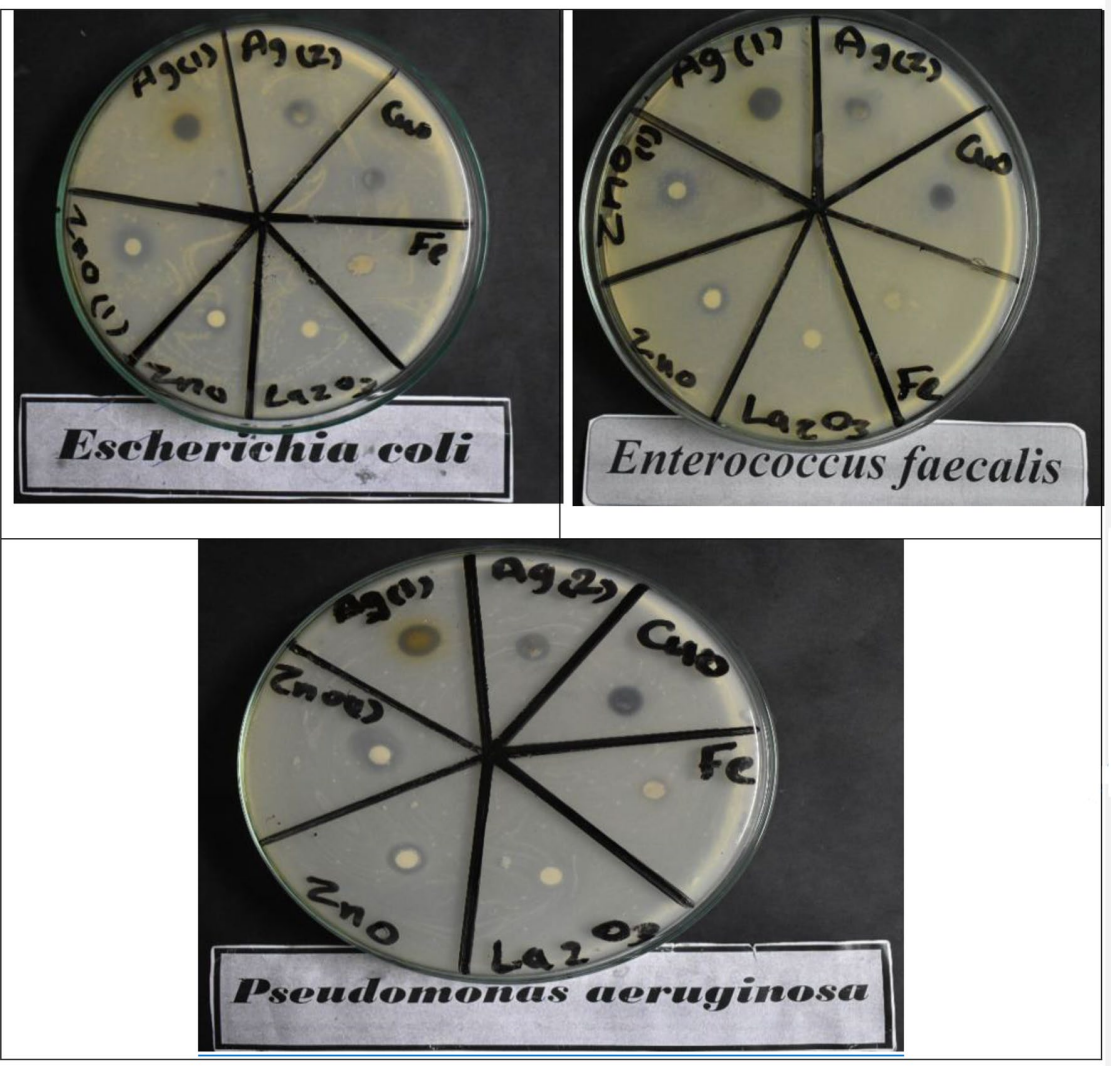
Antibacterial activity (Using *Kirby-Bauer Method*) of metal nanoparticles from aqueous extract of *M. oleifera.*

Table 4 and Fig. 7 showed that tested NPs (La_2_O_3_, CuO, Fe_2_O_3_, Ag, and ZnO) biosynthesized by *M. oleifera* leaves extract exhibited antibacterial activities. All were active against both the G−ve (*E. coli*, *Pseudomonas aeruginosa*, and *Salmonella typhimrium*) and the G + ve bacteria (*B. subtilis*, *Enterococcus faecalis*, and *Staphylococcus aureus*) compared with the standard antibacterial agents used (kanamycin and ampicillin). The antibacterial activity is measured by the diameter of the inhibition zone surrounding the paper discs saturated with the *M. oleifera* NPs. The obtained results revealed that CuONPs recorded the highest activity against *Bacillus cereus* by 24 mm when compared with kanamycin as the standard antibacterial (by 28 mm), However, AgNPs exhibited the highest antibacterial activity against both *Enterococcus faecalis* and *Staphylococcus aureus* by 17 and 15 mm when compared with kanamycin as the antibacterial standard (29 and 25 mm, respectively). Furthermore, ZnONPs recorded the highest antibacterial activity against all tested negative gram bacteria (*Escherichia coli*, *Pseudomonas aeruginosa*, and *Salmonella typhimrium*) by 15, 16, and 14 mm when compared with the tested antibacterial standard (Ampicillin), which exhibits 25, 26, and 28 mm respectively. This suggests that the biosynthesized NPs contained several different antibacterial substances (derived from *Moringa* sp extract) with variable efficiencies and modes of action, which may act synergistically, leading to an increase in the diameter of the inhibition zone. These results may be because of different types, sizes of metal NPs, and their massive counterparts, which result in their mode of action on different bacterial cells.

**Table 4:** Antibacterial activity of different biosynthesized nanoparticles from *M. oleifera*.

Treatments		Inhibition zone diameter (mm / mg sample)					
		Bacterial species					
		G^+^			G^−^		
		Bacillus cereus	Enterococcus faecalis	Staphylococcus aureus	Escherichia coli	Pseudomonas aeruginosa	Salmonella typhimrium
Control : DMSO		0.0	0.0	0.0	0.0	0.0	0.0
Antibacterial agent Standard:	kanamycin	28	29	25	–	–	–
	Ampicillin	–	–	–	25	26	28
Ag		15	17	15	14	14	12
CuO		24	15	12	11	12	12
Fe_2_O_3_		10	11	0.0	10	9	10
La_2_O_3_		10	11	0.0	0.0	10	10
ZnO		14	16	15	15	16	14

G: Gram reaction; Solvent: DMSO .

These results agreed with the results obtained by Almessiere et al*.*^29^; Lansdown^37^; Asgary et al*.*^38^ they reported that silver oxide NPs (Ag_2_O–NPs) have fewer insalubrities and higher surface area to volume ratios than their massive counterparts, resulting in new characteristics. It has a wide range of bactericidal and fungicidal actions, as well as the ability to work with a variety of ligands and macromolecules in the microbial cell. It's commonly used as a covering to prevent microbial infections on medical devices including orthopaedic and cardiovascular implants. Other study by Taghavizadeh Yazdi et al*.*^33^ reported that the biosynthesized AgNPs seemed to exhibit a higher bactericidal and antifungal activity against infective bacteria (*S. aureu* s, *E. coli, P. aeruginosa*) and pathogendic fungi (*Candida* species). Also, the data obtain by Ansari et al*.*^39^ revealed that the formation of pores in the bacteria's cell wall, changes in the permeability of the cell membrane, and the deposition of NPs at these sites are the main causes of bacterial growth suppression by NPs. NPs' antibacterial activity is explained by a variety of processes. The thiol group of electron transport chain enzymes may be disturbed, followed by the adhesion of NPs to the cell wall and membrane of bacteria, resulting in the AgNPs ' inhibitory impact. The negative charge of the microbial cell wall and the positive charge of NPs attract NPs to bacteria. The permeability of the bacterial membrane changes because of this contact, resulting in cell death and disruption.

Another study by Dakal et al*.*^40^ found that there are two potential actions for the effect of NPs on bacterial cells. The uptake of silver ions by bacteria may cause the generation of ATP to be disrupted, causing DNA replication to be disrupted. NPs also cause free radical production, putting the cells under oxidative stress. Apart from that, NPs can disrupt the bacterial cell membrane directly, resulting in cell lysis. Moreover, Van Hengel et al.^41^ reported that nanoparticles can targets bacterial cells via different mechanisms i.e., by altering cell membrane permeability, protein activation, oxidative stress, enzyme activation and gene expression. Due to these unique properties, it becomes difficult for bacteria to develop resistance against NPs. The same authors also concluded that Gum Moringa based NPs not only worked against normal bacteria but also shown considerable activity against resistant bacteria.

## Conclusion

From the obtained results in the present study, it can be concluded that the leaves of *M. oleifera* contain a wide variety of active ingredients, especially reducing agents such as phenolics, flavonoids, and carbohydrates that could serve as antioxidants, antiradicals, and reducing or capping agents in the synthesis of various NPs (La_2_O_3_, CuO, Fe_2_O_3_, Ag, and ZnO). The antioxidant of different biosynthesized metal NPs was dependent on the concentration of extract and incubation time. The obtained results of the tests of nanoparticles as cytotoxic effect conclude that acceptable potency against T47D and A549 cell lines with IC_50_ ranged from 38 to 210 μg/mL and 26 to 115 μg/mL, respectively. However, HepG2 and Wi38 cell lines showed relatively higher resistance against all tested NPs when compared with Doxorubicin. Moreover, the antibacterial results revealed that AgNPs exhibited the highest antibacterial activity against both *Enterococcus faecalis* and *Staphylococcus aureus* when compared with kanamycin as an antibacterial standard. Furthermore, ZnONPs recorded the highest antibacterial activity against all tested gram-negative bacteria (*Escherichia coli*, *Pseudomonas aeruginosa*, and *Salmonella typhimrium*). From this obtained data, we recommend using the biosynthesized NPs in the fields of pharmacological and medicinal research after determining the safe dose for each tested metal NPs.

## Data Availability

They are available as Supporting information.

## Abbreviations

BHT: Butylated hydroxyl toluene
ATP: Adenosine tri-phospahate
DPPH: Diphenyl picryl hydrazyl
NPs: Nanoparticles
ROS: Reactive oxygen species
SI: Selective index
MTT: 3-(4,5-Dimethylthiazol-2-yl)-2,5-diphenyltetrazolium bromide
